# An Exuberant Case of Ulceronodular-Rupioid (Malignant) Syphilis in an HIV Patient: A Proposal for New Diagnostic Criteria

**DOI:** 10.3390/idr16030038

**Published:** 2024-06-06

**Authors:** Dennys Jimenez, Marian Santillan Rabe, Apeksha N. Agarwal, Scott R. Dalton, Gregory M. Anstead

**Affiliations:** 1 Department of Medicine, Division of Infectious Diseases, University of Texas Health San Antonio, 7703 Floyd Curl Drive, San Antonio, TX 78229, USA; jimenezhernd@uthscsa.edu; 2 Family-Focused AIDS Clinical Treatment Services Clinic, University Health System, 903 W. Martin St., San Antonio, TX 78207, USA; marian.santillanrabe@uhtx.com; 3 Department of Pathology, University of Texas Health San Antonio, 7703 Floyd Curl Drive, San Antonio, TX 78229, USA; apekshan0210@gmail.com; 4 Sagis Diagnostics, PLLC, 4131 Directors Row, Houston, TX 77092, USA; sdalton@sagisdx.com; 5 Medical Service, Division of Infectious Diseases, South Texas Veterans Healthcare System, 7400 Merton Minter Blvd., San Antonio, TX 78229, USA

**Keywords:** malignant syphilis, human immunodeficiency virus, lues maligna, *Treponema pallidum*, ulceronodular syphilis, rupioid syphilis

## Abstract

We report the case of a 28-year-old male with uncontrolled human immunodeficiency virus (HIV) infection who presented with extensive ulcerated lesions with dark lamellated crusting on his face, torso, and limbs. The patient had a rapid plasma reagin (RPR) titer of 1:512, indicative of syphilis. A skin biopsy revealed granulomata surrounded by lymphocytes, histiocytes, and plasma cells, with spirochetes visible on immunohistochemical staining. The patient’s rash resolved with hyperpigmented scarring after penicillin and doxycycline treatment. This severe form of secondary syphilis has been termed malignant syphilis, lues maligna, ulceronodular syphilis, or rupioid syphilis. We propose a single descriptive name for this entity, ulceronodular-rupioid syphilis. In 1969, Fisher proposed criteria for malignant syphilis based on lesion appearance, histopathologic findings, high RPR values, and rapid response to treatment. We found that the Fisher criteria were imprecise with respect to specific histopathologic findings, the quantitation of RPR values, and what constitutes rapid response to treatment. Thus, we examined an additional 74 cases from the literature and propose new diagnostic criteria based on rash appearance, histopathologic characteristics, non-treponemal and treponemal test positivity, and response to therapy. We also found that uncontrolled viremia, and not a low CD4 count, is a major risk factor for ulceronodular-rupioid syphilis in HIV patients.

## 1. Introduction

Syphilis is a sexually transmitted infection caused by the spirochetal bacterium *Treponema pallidum* that can cause serious consequences to health without prompt treatment. It proceeds through four stages (primary, secondary, latent, and tertiary) that may be progressive if untreated, and these stages may sometimes overlap [1]. Primary syphilis manifests as a chancre, a painless ulcer that usually occurs in genital, anal, or oral sites, that may go unnoticed. Around 1–2 months after the chancre spontaneously resolves, patients become bacteremic with *T. pallidum*, initiating secondary syphilis. The rash of secondary syphilis is typically its most characteristic finding; it is usually a diffuse, erythematous macular or papular eruption that involves the trunk and extremities, including the palms and soles; however, there are multiple variations of this presentation [2,3]. Also, the secondary stage may be accompanied by constitutional symptoms, lymphadenopathy, and deeper organ involvement (meningitis, ocular disease, osteitis, mucosal ulceration, hepatitis, and nephritis) [1,4]. If the patient recovers from the secondary stage, the infection enters an asymptomatic latent period that may last for years. Tertiary syphilis may occur years after the initial infection and primarily affects the cardiovascular and nervous systems [1].

In most cases, the rash of secondary syphilis is transient and resolves in the absence of antibiotic therapy. A severe variant of secondary syphilis, which manifests as multiple thickened, lamellated (rupioid) plaques and/or ulcerative and necrotic lesions, has been termed ulceronodular syphilis, malignant syphilis, lues maligna, or rupioid syphilis [5]. This dramatic presentation is usually seen in patients with human immunodeficiency virus (HIV) infection or other immunocompromising conditions.

The purpose of the current report is to present the dramatic appearance of the rash in a patient with malignant syphilis (which henceforth we will refer to as ulceronodular-rupioid syphilis (UNRS)), its histopathologic characteristics, and the sequelae of the rash 15 months after treatment. In 1969, Fisher and coworkers proposed criteria for malignant syphilis based on lesion appearance, histopathologic findings, high RPR values, rapid response to treatment, and the occurrence of the Jarisch–Herxheimer reaction (JHR) [6]. These criteria have been repeatedly cited in the medical literature for over 50 years. In the course of evaluating this patient, we found that the Fisher criteria were imprecise with respect to specific histopathologic findings, the quantitation of RPR values, and what constitutes rapid response to treatment. Thus, we examined the features of an additional 74 cases from the literature and propose new diagnostic criteria based on rash appearance, histopathologic characteristics, non-treponemal and treponemal test positivity, and response to therapy. We also found that uncontrolled viral load is a major risk factor for UNRS in HIV patients.

## 2. Methods

To create a database of cases from which to establish diagnostic criteria, the PubMed database was searched using the terms “malignant syphilis OR lues maligna OR rupioid syphilis OR ulceronodular syphilis” for papers published in English or Spanish between January 2019 and June 2023; 29 cases were found (Appendix A, Table A1). We compared this series of 29 cases (plus the current case) to a series of 45 cases reviewed by Wibisono et al. based on reports from 2014 to 2018 [7] with respect to demographics, risk factors, time to presentation, syphilis serologic titers, histopathologic features, and response to therapy. Based on the characteristics of these 75 patients, a new set of diagnostic criteria for UNRS were derived.

## 3. Case

The patient is a 28-year-old African American male with a past medical history of HIV infection, hypertension, asthma, morbid obesity, anxiety disorder, and syphilis. He was last seen in clinic 18 months prior to his current visit. He has a history of medication non-adherence. At his prior visit, he had a CD4 count of 491 cells/µL (19%) and an HIV RNA viral load of 17,000 copies/mL and was prescribed abacavir-dolutegravir-lamivudine. At the current visit, the patient reported a rash of 3 months duration that started as slightly tender red to purple papules on his trunk, face, and upper extremities that progressively increased in number. The papules thickened, eventually cracking, and some were draining a malodorous fluid. He reported a 6.8 kg weight loss for the same period because the multiple lesions on lips made it difficult for him to open his mouth, but he denied intra-oral lesions. The patient was admitted to the hospital for evaluation of this diffuse dermatitis and a chronic dry cough. He denied fever, chills, neurologic symptoms, insect bites, animal exposures, or contact with persons with tuberculosis. Three years prior, he was treated by the local health department for late latent syphilis with three weekly injections of intramuscular (IM) benzathine penicillin.

**Figure 1:**
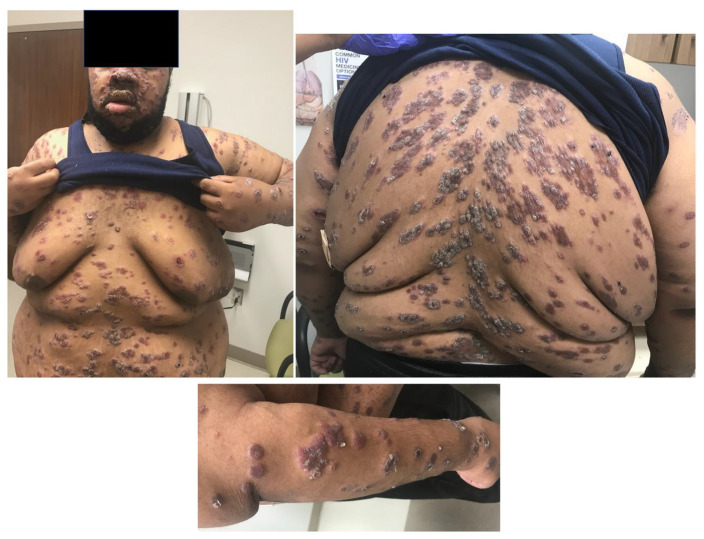
Multiple nodular, scaly, and ulcerative lesions of the trunk, face, and right arm.

The initial differential diagnosis for the patient’s florid rash included syphilis or a systemic mycosis. On admission, he was febrile up to 38.4 °C, with tachycardia (pulse 110–126 beats per minute). Physical exam revealed widely disseminated firm violaceous papules and nodules mixed with crusted ulcerated plaques, some with malodorous serosanguinous drainage, that involved the face, nasal vestibules, neck, chest, back, abdomen, bilateral upper and lower extremities, and the groin, but spared the oral mucosa and genitals. Ulcerative lesions were noted on the left palm and plantar surfaces of both feet. The lesions on the back displayed a configuration similar to pityriasis rosea (Figure 1). Laboratory evaluation showed an elevated C-reactive protein at 38 mg/dL (reference range (RR) < 10 mg/dL) and normocytic anemia (hemoglobin 9.2 mg/dL (RR 12.8–17.1 mg/dL), which was suspected to be due to uncontrolled HIV infection. Testing for systemic mycoses (*Histoplasma* urine antigen, *Coccidioides* IgM/IgG, fungal serologic panel), blood cultures, latent tuberculosis (interferon-gamma release assay), hepatitis C IgG, and sexually transmitted diseases (gonorrhea, chlamydia, syphilis, herpes simplex) was obtained. A skin punch biopsy was performed, and bacterial, AFB and fungal cultures were collected. Due to his respiratory symptoms, a computerized tomography of the chest was performed, which showed axillary and retropectoral lymphadenopathy, but no abnormal lung findings. He was started on intravenous cefepime and vancomycin for coverage of bacterial skin infections. A culture of one of the draining lesions grew methicillin-resistant *Staphylococcus aureus*. Three days later, his antibiotics were changed to oral doxycycline 100 mg twice daily and cephalexin 500 mg four times a day to complete 7 days of treatment of staphylococcal and/or streptococcal infection of the multiple draining skin lesions.

**Figure 2:**
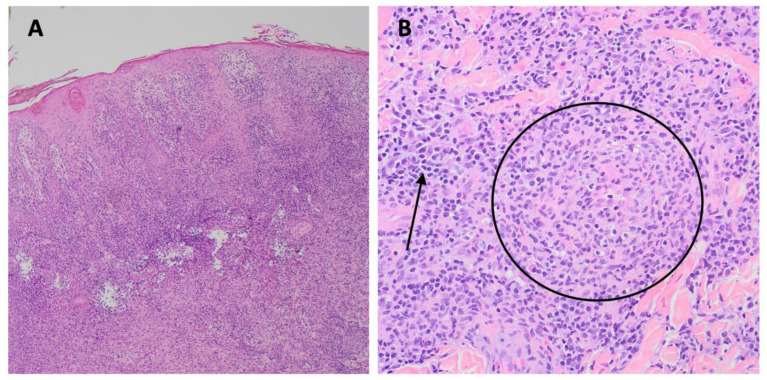
(**A**). Photomicrograph of skin biopsy specimen showing a psoriasiform lichenoid infiltrate with dense dermal inflammation (Hematoxylin and Eosin stain (H&E), ×40). (**B**). Photomicrograph of skin biopsy specimen showing a granuloma (circle) with surrounding lymphocytes and plasma cells (arrow), (H&E, ×400).

**Figure 3:**
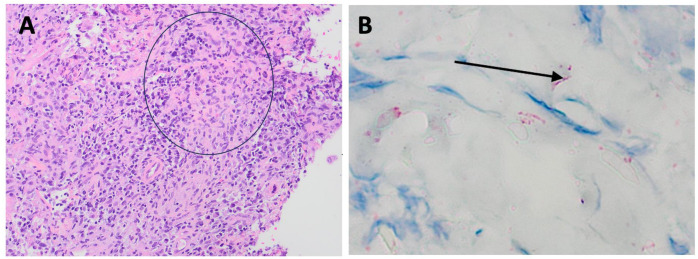
(**A**). Photomicrograph of skin biopsy specimen showing mixed dermal infiltrate of neutrophils, histiocytes, lymphocytes, and plasma cells (H&E, ×200). (**B**). Photomicrograph of skin biopsy showing spirochetes highlighted by red chromogen spirochete immunohistochemical stain (arrow), ×1000.

The patient had a positive specific treponemal antibody test and a rapid plasma reagin (RPR) titer of 1:512. The skin biopsy revealed a lichenoid psoriasiform pattern with granulomata surrounded by lymphocytes, histiocytes, and plasma cells, with visible spirochetes on immunohistochemical staining (Figure 2 and Figure 3). He was given one dose of intravenous penicillin G (2.5 million units) and subsequently had a fever of 38.9 °C, which was suspected to be a Jarisch–Herxheimer reaction. No organisms were identified in the biopsy specimen using Fite, acid fast, Gomori’s methenamine silver, and periodic acid Schiff stains, and an immunohistochemical stain for human herpesvirus-8 was negative. The blood cultures showed no growth at 5 days. Fungal and acid-fast bacilli cultures of the biopsy specimen showed no growth after 4 and 6 weeks, respectively. Gonorrhea and chlamydia were detected by nucleic acid amplification tests on a rectal swab, and the patient was treated with one dose of 1 g of oral azithromycin. His CD4 count was 411 cells/µL (21%), and his HIV viral load was 35,200 copies/mL. The patient’s antiretroviral therapy was switched to bictegravir-tenofovir alafenamide-emtricitabine due to the association of abacavir with an increased risk for cardiovascular events [8]. Other discharge medications were cephalexin and doxycycline.

**Figure 4:**
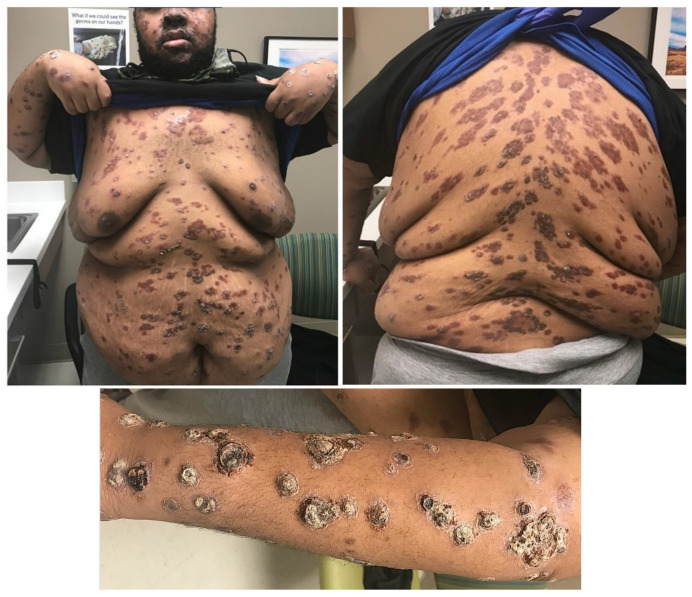
Appearance of the rash one week after treatment with intramuscular benzathine penicillin and doxycycline. The truncal and facial lesions have become less nodular and scaly, but the lesions on the left arm still clearly demonstrate a rupioid appearance.

**Figure 5:**
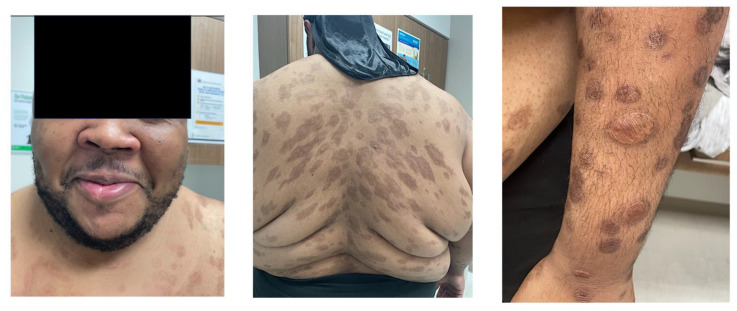
Appearance of the lesions about 15 months after treatment.

The patient was seen in outpatient clinic 7 days after hospital discharge, and the rash of the face, right upper extremity, and torso had partially regressed (Figure 4). Nevertheless, he still had a persistent ulceronodular rash with crusting of his left arm. He reported adherence with the bictegravir-tenofovir alafenamide-emtricitabine, cephalexin, and doxycycline. He was given intramuscular benzathine penicillin 2.4 million units weekly for two doses due to concerns of late latent syphilis, and doxycycline was extended an additional 7 days for a total of 17 days to treat secondary staphylococcal skin infection and rectal chlamydia. Eight weeks later, his RPR decreased to 1:128, and his HIV viral load dropped to 21 copies/mL. He was subsequently seen for two additional clinic visits but was then lost to follow-up for 1 year. He returned to clinic about 15 months after his initial presentation, and the plaques and ulcers on the trunk and extremities were completely healed, but there was scarring with post-inflammatory hyperpigmentary alteration at the sites of prior involvement. Fortunately, the lesions on the face healed with minimal scarring (Figure 5). The follow-up RPR level 15 months after completing treatment had decreased to 1:32 (a 16-fold drop).

## 4. Results: Case Series Summary

To determine the appropriateness of the Fisher criteria [6], we assembled a series of 29 cases published from 2019 to 2023 (in addition to the current case; see Appendix A, Table A1). Fisher’s original case is denoted in Table A1 as case Fi [6]. We also re-examined the series of 45 cases reported by Wibisono et al. based on studies published from 2014 to 2018 [7].

## 5. Discussion

### 5.1. Nomenclature

The nodular, ulcerative, and necrotic lesions with lamellated plaques observed in this syphilis patient have been termed lues maligna, syphilis maligna praecox, malignant syphilis, nodulo-ulcerative syphilis, ulceronodular syphilis, syphiloderma ulcerativum, and rupioid syphilis [9,10,11]. Rupioid refers to “thick, dark, lamellate, and adherent crusts… that may resemble an oyster shell” [12]. It is not a malignancy but was given this name based on its severe clinical features [13]. We advocate a single name for this condition, ulceronodular-rupioid syphilis (UNRS), which describes the lesions characteristic of this condition and avoids archaic terms such as lues and praecox and terms that are better applied to neoplastic processes, such as malignant and maligna. 

### 5.2. Historical Perspective

This severe manifestation of secondary syphilis was first described by French physician Pierre-Antoine-Ernest Bazin in 1859 [14]. Ulceronodular-rupioid syphilis was not fully accepted as a manifestation of secondary syphilis until the works of Neisser [15] and Haslund [16] in 1897 [7,17,18]. Few cases of UNRS were described before the HIV pandemic. Currently, most cases of UNRS are reported in HIV-positive men who have sex with men (MSM).

In the bygone era of Neisser, in which there were many cases of tertiary syphilis, Neisser concluded that malignant syphilis was a manifestation of secondary syphilis, based on its short incubation period [15]. The lesions of UNRS differ from those of tertiary syphilis by (1) being multiple rather than solitary or few; (2) round or oval shape rather than arciform; (3) the presence of central ulceration with peripheral extension; and (4) the presence of lamellar crusting [6].

### 5.3. Description and Evolution of the Rash

The rashes of secondary syphilis may be urticarial, macular, maculopapular, papular, pustular, and/or nodular [2]. The lesions of UNRS start as papules that evolve into pustules that undergo central necrosis, resulting in ulcers that scab over with rupioid crusts [19]. The deep ulceration and lamellar crusting are what distinguishes UNRS from other rashes of secondary syphilis. The lesions of UNRS primarily occur on the face, trunk, and limbs [7]; it rarely affects oral mucosa and palmoplantar areas, unlike the more typical mucocutaneous presentations of secondary syphilis [20]. The cutaneous sequelae of treated UNRS vary from minimal to hypo- or hyperpigmented macules with or without scarring (Appendix A, Table A1). Our patient suffered extensive scarring with post-inflammatory hyperpigmentary alteration that fortunately spared his face. 

### 5.4. Differential Diagnosis 

Cutaneous disorders are often the hallmarks of uncontrolled HIV infection, and the differential diagnosis is broad, including cutaneous lymphomas, pityriasis lichenoides, lymphomatoid papulosis, bacillary angiomatosis, mycosis fungoides, disseminated herpes simplex/varicella infection, ecthyma gangrenosum, Reiter syndrome, vasculitis, Mpox, leishmaniasis, psoriasis, pityriasis rubra pilaris, Norwegian scabies, prurigo nodularis, and various mycoses (cryptococcosis, histoplasmosis, coccidioidomycosis, and sporotrichosis) [9,21,22]. Furthermore, these cutaneous disorders may also have superimposed bacterial infection, as in our patient.

Because patients with uncontrolled HIV infection often have multiple conditions occurring simultaneously, to definitively diagnose a case of UNRS, it is necessary to perform syphilis serologic testing and obtain a skin biopsy [7]. In biopsy specimens of UNRS, there is a relative paucity of spirochetes [10]. Based on the reviewed cases, immunohistochemical staining detects spirochetes in about 80% of cases of UNRS, whereas silver staining is positive in only 40–50% of cases (Table 1). Previously, the sensitivity of immunohistochemical staining and silver staining in the detection of spirochetes in skin biopsy specimens of patients with secondary syphilis has been reported to be 71–87%, and 33–70%, respectively [3,23].

Thus, spirochetes are typically present in UNRS lesions, which is crucial to differentiate these lesions from syphilitic gummas [11]. In both the Wibisono and our series, cases with a biopsy specimen showing negative staining were considered to be UNRS because of consistent dermatologic presentation, positive non-treponemal and treponemal tests, and suggestive histopathologic findings (dermal infiltrate with plasma cells and lymphocytes, sometimes with non-caseating granulomas and/or vasculitis) [7].

### 5.5. Epidemiology, Risk Factors, and Pathogenesis of Ulceronodular-Rupioid Syphilis 

In 1897, Haslund found that in the pre-HIV era, the prevalence of UNRS was 0.36% of all syphilis cases [16]. Before the HIV-1 epidemic, only 14 cases of UNRS were reported in English from the 1900s through the early 1980s [7,24]. In the HIV era, a multicenter retrospective study conducted in Germany found that 1.3% (151/11,368) of HIV-infected individuals had syphilis, of which 7.3% (11/151) had UNRS. HIV patients with syphilis were 60 times more likely to present with UNRS compared with non-HIV patients [13]. In one series of 332 syphilis cases, UNRS was diagnosed in 2% of the 202 syphilis patients infected with HIV, with zero cases in the non-HIV patients [22]. In Table 1, we compare the general characteristics of the patients with UNRS in the case series of Wibisono [7] with our case series of 30 patients (Appendix A, Table A1) to ascertain risk factors for the presentation of UNRS. 

In our series, the mean age of the patients was 35.7 years, with a range of 16–61. One patient in the Wibisono series was 86 years old [25], and so immunosenescence may have been the risk factor in that case. For the 30 patients in our series, 63% were male, compared to 84% in the Wibisono series [7]. A preponderance of males is expected, considering the high incidence of syphilis in the MSM population [26]. For both series, the time to presentation varied from 1 to 36 weeks, with a mean of 6 to 7 weeks, and a modal duration of 4 weeks. This large range of the duration of illness prior to presentation may affect both the appearance of the rash and the histopathologic findings.

The percentage of patients with HIV infection was 73% in the Wibisono series [7] and 57% in our series. Considering both series, the CD4 count range in HIV patients was 23–1294 cells/µL. In the Wibisono series [7] and our series, the mean CD4 counts were 397 and 291 cells/µL, respectively, with 69% of the HIV patients in the combined series having a CD4 count above 200 cells/µL, the level at which significant impairment of cellular immunity occurs. 

Schöfer and coworkers compared the mean CD4 counts of 44 HIV-infected syphilis patients with typical maculopapular rashes versus 11 patients with UNRS [13]. The latter group did have a lower mean CD4 count, of 307 cells/µL (*SD* = 140), than the former group (mean 470 cells/µL; *SD* = 355). However, using a one-tailed *t*-test, this difference in CD4 cell counts between the two groups was not statistically significant (*p* = 0.07) [27].

However, we found that 81.5% of the HIV patients with UNRS in the Wibisono series [7] and 88.2% in our series had uncontrolled viremia (Appendix A, Table A1). Considering the wide range and relatively high mean of CD4 counts for patients in both series and the data of Schöfer and colleagues, overt CD4 cell deficiency is not the only factor promoting UNRS in HIV patients. Uncontrolled HIV viremia may be a significant risk factor predisposing to UNRS. 

The host’s immune status determines the pathogenesis and course of syphilis through its various stages. A robust delayed-type hypersensitivity (DTH) response, mediated by CD4 cells, is crucial to the control of syphilis. Humoral immunity and CD8 cytotoxic T cells are ineffective in clearing the infection and preventing its progression. In DTH, an expanding population of antigen-specific CD4 cells release Th1 cytokines that recruit and activate macrophages at the site of infection, resulting in phagocytosis and pathogen killing. Persistent localized antigenic challenge produces excessive inflammation, with resultant plasma cell infiltration, granuloma formation, and tissue destruction [3]. In their study of the effects of antiretroviral treatment on CD4 responses in treatment-naive HIV-infected patients, Wendland and coworkers found that suppression of viremia is necessary for full restoration of DTH. The mechanism underlying this phenomenon may be interference by HIV virions or soluble gp160 with chemokine receptors that are necessary for the recruitment of inflammatory cells [28]. This may explain the importance of HIV viremia in the predisposition to UNRS.

The pathogenesis of UNRS in HIV patients may differ from the typical maculopapular rash of secondary syphilis in an immunocompetent person because CD4 T-cell depletion leads to increased tissue infiltration and activation of cytotoxic T cells and neutrophils [7,29]. Furthermore, there is defective CD4 cell function in the setting of uncontrolled viremia.

However, Zhu and colleagues have reported a series of 26 UNRS patients treated at a sexually transmitted infection clinic in China from 2008 to 2018 in which only 30.7% were HIV-positive [30]. In the Zhu study, seven of the HIV patients with UNRS had available CD4 counts; the range was 50–518 cells/µL, with a mean of 337 cells/µL; 71.4% had a CD4 count greater than 200 cells/µL [30]. Information on HIV viremia was not available. These results are in accord with the Wibisono and our series, in which most HIV patients with UNRS had a CD4 count above 200 cells/µL. 

In addition to patients with HIV infection, UNRS has also been observed in patients with alcoholism/alcoholic hepatitis, malnutrition, uncontrolled diabetes mellitus, intravenous drug use, psoriasis, chronic kidney disease, and advanced age [7,31]. In our series (Appendix A, Table A1), other observed risk factors were juvenile rheumatoid arthritis under immunosuppressive treatment (methotrexate, prednisone, and an interleukin-6 receptor blocker) and Crohn’s disease (on a tumor necrosis factor-alpha blocker). However, 5/45 patients (11.1%) in the Wibisono series and 5/30 patients (16.7%) in our series had no apparent risk factors for UNRS [7]. Although infection by a hypervirulent *Treponema pallidum* strain has been proposed to be a possible cause of UNRS [32], there is no evidence to support this supposition [33]. To quote Neisser’s 1897 paper: “…it is quite established in cases of malignant syphilis that the source need not been a malignant case and also the individual suffering from malignant disease need not bring about a malignant case in another if infection occurs. We must conclude … that malignancy is due to the peculiar susceptibility of the affected person… [15].” 

In the Zhu series [30], the CD4 counts of 15 non-HIV patients with UNRS were examined. They found a mean CD4 count of 740 cells/µL, with a range of 275–1069 cells/µL. Again, this indicates an immunodeficiency apart from low CD4 count that predisposes to the development of UNRS. Sammet and Draenert reported an HIV patient who developed UNRS three consecutive times (likely re-infections) despite the start of antiretroviral therapy [34]. Likewise, Wang and coworkers reported an HIV patient who had two episodes of UNRS, separated by 13 months, despite receiving ART after the first episode [19]. Thus, these two patients, despite improving CD4 counts and suppression of HIV viral load, had another unknown immunologic predisposition to develop UNRS, such as a specific genetic polymorphism that affects the expression of particular cytokines, chemokines, or their receptors [35,36].

### 5.6. Syphilis Complications Associated with Ulceronodular-Rupioid Syphilis

In our series of 30 patients, other syphilis manifestations included uveitis (four cases); neurosyphilis (four cases); condyloma lata (two cases); osteitis (one case): papillitis (one case): mucus patch (one case); and splenitis (one case) (Appendix A, Table A1). We re-examined the 45 patients in the Wibisono series, and there were relatively few syphilis complications: vitritis (two cases [37,38]); keratitis (one case [37]); neurosyphilis (two cases [22,39]); condyloma lata (one case [22]); mucus patch (one case [25]); osteitis (two cases [32,40]); orchitis (one case [40]; pulmonary nodules (one case [40]); and uveitis (one case [22]). In both the Wibisono and our series, some patients had multiple syphilis manifestations occurring simultaneously. In an analysis of 135 cases, Zhu concluded that a much higher proportion of patients with UNRS present with concurrent neurosyphilis (30%) as compared to secondary syphilis patients without UNRS (13%) [30]. Thus, the clinician should be alert to possible neurosyphilis and ocular complications in patients with UNRS, because these cases will require more intensive treatment.

### 5.7. Treatment of Ulceronodular-Rupioid Syphilis 

An unanswered question is how best to treat UNRS that does not have concurrent neurologic or ocular disease. As a manifestation of secondary syphilis, US Centers for Disease Control and Prevention guidelines [41] state that the preferred treatment is a single dose of intramuscular benzathine penicillin (BP), as previously advocated [42]. However, most practitioners have used weekly intramuscular benzathine penicillin (IM BP) × 3 doses. In the Wibisono series, 21/26 (81%) received three doses of IM BP, whereas only 5/26 (29%) received a single dose of IM BP; all the patients with known outcomes showed improvement or resolution of the rash with either dosing regimen [7]. In our series, 18 cases were treated with IM BP; 13/18 (72%) received three weekly injections, with all patients improving. In the three cases with a known outcome treated with a single dose of IM BP, all showed improvement or resolution. All nine cases with a known outcome from the two series that received single-dose IM BP treatment showed improvement or resolution. Thus, it is likely that in the absence of neurologic or ocular involvement, a single dose of IM BP is sufficient treatment. In the Zhu series, 10 patients received IM BP weekly for 2 weeks, with resolution in all cases [30]. Our patient was treated with two doses of weekly IM BP and doxycycline to treat concurrent staphylococcal skin and soft tissue infection and rectal chlamydia. Oral tetracyclines have also been used with good clinical response in the treatment of UNRS [6,7,10,19,32,43]. 

### 5.8. Previous Diagnostic Criteria of Ulceronodular-Rupioid Syphilis 

Many of the papers on UNRS cite the work of Fisher et al. from the pre-HIV era (1969) for the diagnostic criteria of this affliction [6]. Fisher and colleagues, in turn, reference an 1897 paper by venereologist Albert L. Neisser (Table 2). However, both sets of criteria are problematic. For the Neisser criteria, the incubation period is usually unknown unless there is a single specific sexual encounter. Also, mucous membrane involvement is seldom reported in recent cases of UNRS. The lesion description by Neisser is consistent with recent cases. However, Neisser did not incorporate any histopathologic or serologic characteristics into his criteria [15].

### 5.9. A High Serological Titer as a Diagnostic Criterion 

A “high” serologic titer is one of Fisher’s criteria, but this is not useful because high was not defined. To determine the typical levels of RPR and Venereal Disease Research Laboratory (VDRL) serologic titer elevations that occur in UNRS, we examined the data of the patients in the Wibisono series [7], our series, and the Zhu series [30] (Table 3). RPR and VDRL titers need to be evaluated separately, because RPR titers tend to be higher than VDRL titers for the same patient [44].

In the Wibisono series, 17/21 cases (81%) had an RPR titer of 1:64 or higher [7]. However, there were two cases with an RPR value of only 1:4 that had dermatologic and histopathologic characteristics consistent with UNRS. Thirteen of 14 (93%) patients in the Wibisono series had a VDRL titer of 1:64 or higher, but one patient had a titer of only 1:8 [7]. In our series, 16 of 21 patients (76%) had an RPR titer of 1:64 or higher; three patients had an RPR titer of 1:16. In the Zhu series, 24/26 (92%) had an RPR of 1:64 or higher [30]. Thus, for the three series, 57/68 patients (84%) had an RPR of 1:64 or higher. Eleven cases in the current series had published VDRL titers; in 7/8 (88%), titers were 1:32 or higher. Thus, we conclude that in UNRS, 84% of patients have an RPR titer of 1:64 or higher, and >90% have VDRL titers of 1:32 or higher. Occasionally, a detectable RPR titer is not observed in a patient with UNRS. This is likely due to the prozone phenomenon [45], in which there is a high concentration of antibody in the tested serum. This prevents the flocculation reaction required to attain a positive RPR or VDRL result [33]. Co-infection with HIV is a risk factor for the prozone effect in syphilis diagnosis [45].

Also, there may also be false positive non-treponemal tests. The minimum RPR titer during secondary syphilis that most likely indicates a true positive is 1:8 [46]. Furthermore, high RPR titers need to be confirmed as syphilis by a treponemal test because high false positive non-treponemal titers can occur in intravenous drug abusers and in HIV infection [46,47]. False negative non-treponemal tests can also occur, especially in HIV-positive and other immunocompromised patients [3,48]. 

### 5.10. Rejection of the Jarisch–Herxheimer Reaction (JHR) as a Diagnostic Criterion

In secondary syphilis, the JHR may present with fever, chills, headache, myalgias, and hypotension within hours after initial antibiotic administration. The JHR usually resolves without treatment within 24 h [7]. Fisher and coworkers [6] used the occurrence of the JHR as one of their diagnostic criteria. In the Wibisono series, only 24% had a reported JHR [7]. In our series of 30 patients, eight papers did not mention the JHR, five groups used corticosteroid administration to prevent the JHR and were excluded from our analysis, and JHR positivity was established in only five of the 17 patients in which the presence or absence of a JHR was documented (29%). In the Zhu series, 72% experienced the JHR [30]. Thus, with such a wide range of positivity rates, the occurrence of the JHR is rejected as a diagnostic criterion of UNRS.

### 5.11. Rapid Response to Treatment as a Diagnostic Criterion

Fisher et al. used “rapid response” to therapy as a diagnostic criterion, but the terms “rapid” and “response” were not defined. Based on the literature, most patients with UNRS were assessed in a non-standardized time frame after treatment, so the best that can be said is response or non-response of the lesions weeks to months after treatment. Serologic assessment 6 to 12 months after treatment is not available for most of the cases in our series and for the Wibisono patients [7]. In all 41 cases of the Wibisono series with a known outcome, the treatment was successful [7]. In our series, 25 cases had a known outcome, and all patients showed improvement or resolution (Appendix A, Table A1). In the Zhu series, all 26 patients had a successful clinical and serologic outcome at 3 months [30]. Nevertheless, lesions with secondary pyogenic infection may not respond without other appropriate antibiotic therapy. 

### 5.12. Histopathologic Criteria for Ulceronodular-Rupioid Syphilis

Fisher and coworkers used “compatible gross microscopic morphology” as one of their criteria of UNRS without defining this term [6]. One difficulty in establishing histopathologic criteria for UNRS is that the skin lesions are pleomorphic and patients present at variable times during the course of their illness. In our series, the duration of illness before presentation ranged from 2 to 20 weeks (Table A1 and Table 1), with a mean of 5.7 weeks; in the Wibisono series, the duration of illness prior to presentation varied from 1 to 36 weeks, with a mean of 7.1 weeks [7]. Thus, due to the differing durations of time prior to presentation, the lesions are in different stages of evolution, so the histopathologic findings may differ between them. In Fisher’s case (Case Fi, Appendix A, Table A1), the duration of illness prior to presentation was 20 weeks [6], which may account for the extensive vascular involvement seen in that case compared to most cases of UNRS. A second problem is that in HIV patients, the histopathologic appearance of a condition may vary according to the CD4 count.

Another problem with defining exact histopathologic criteria for UNRS is that the rashes of secondary syphilis display “an enormous diversity of histological features” with no single diagnostic feature, and the only definitive test is identifying the organism with staining or microscopic techniques [49]. The most common histopathological features of secondary syphilis are a psoriasiform-lichenoid pattern with superficial and deep perivascular and interstitial cellular infiltrate of lymphocytes, plasma cells, and histiocytes (a glossary of histopathologic terms is provided in Appendix B). In early lesions of secondary syphilis, a lichenoid pattern is seen, with lymphocytes predominating, but after a few weeks, the epidermis becomes more psoriasiform, and plasma cells and histiocytes predominate in the superficial and deep perivascular infiltrate [31]. In the typical maculopapular rash of secondary syphilis, the vasculitis is self-limited. However, in UNRS, an obliterative necrotizing vasculitis may be observed, which results in the ulcers and rupioid crusts [6]. By contrast, the gummas of tertiary syphilis are nodules with necrotic foci, surrounded by epithelioid cells and occasional giant cells, in turn enveloped by a lymphoplasmacytic infiltrate and encased by a layer of fibrosis. Spirochetes may be seen in gummas, but they are extremely sparse [50]. 

The histopathologic findings in our case patient were lichenoid psoriasiform dermatitis with an infiltrate of primarily lymphocytes, plasma cells, and histiocytes. Granulomas were present, and spirochetes were seen by immunostaining. The biopsied lesion was likely not secondarily infected with *S. aureus* or *Streptococcus* species because the presence of these organisms would have elicited a prominent neutrophilic response [51,52].

In the Wibisono series [7] and in our series, 35 and 28 cases had reported histopathologic findings, respectively (Table A1 and Table 4). From Table 4, the most common findings were plasma cell and lymphohistiocytic dermal infiltrates, which were observed in 82.5% and 71.4% of cases, respectively. Neutrophilic infiltrates were less commonly described (15.9% of cases). Vascular involvement and non-caseating granuloma formation were noted in 39.7 and 27.0% of patients, respectively. Giant cells were rarely observed (6.3% of cases). Although an abundance of plasma cells is a common finding in UNRS, occasionally the plasma cell infiltrate may be scant, and histologically, the condition may resemble cutaneous T-cell lymphoma [53]. 

### 5.13. Revised Diagnostic Criteria for Ulceronodular-Rupioid Syphilis

Based on the analyses above, we propose the following criteria for the diagnosis of UNRS:

(1) **Dermatologic.** Round or oval pleomorphic skin lesions: papulopustules, nodules, ulcerations, ulcers with brown-black rupioid crusts, and healing lesions (same as Neisser [15]).
(2) **Histopathologic.** Typically, there is a dermal infiltrate of lymphocytes, histiocytes, and plasma cells, often with vascular involvement and/or granuloma formation. Immunohistochemical staining provides confirmation of the presence of spirochetes in about 80% of cases.
(3) **Serologic.** A positive RPR or VDRL titer, with RPR and VDRL titers of at least 1:8 and a positive treponemal test. Eighty-four percent of UNRS patients have an RPR titer of 1:64 or higher, and >90% have VDRL titers of 1:32 or higher.
(4) **Response to Therapy.** Improvement or resolution of the dermatologic manifestations (within weeks to months) with standard treatments for uncomplicated secondary syphilis, either intramuscular benzathine penicillin or doxycycline.

Ulceronodular-rupioid syphilis may be accompanied by pulmonary, cardiovascular, neurologic, gastrointestinal, osseous, otic, ocular, and renal complications. Karanfilian and coworkers have proposed that the case definition should include these organ manifestations [31]. However, both in the Wibisono series [7] and in our current series, internal manifestations of syphilis (ocular syphilis, neurosyphilis, osteitis, pulmonary nodules, and splenic abscess) were documented but were uncommon (Table A1). Certainly, the clinician needs to be cognizant that other syphilis complications may occur in UNRS, but these complications need not be part of the diagnostic criteria.

## 6. Conclusions

Since 2000, the case rate of primary and secondary syphilis has increased 5-fold in the United States [54]. In 2020, the highest rate of reported cases of primary and secondary syphilis was among non-Hispanic Black or African American persons [55]. These disparities are associated with the well-documented barriers to access to health care in minority populations, as well as differences in social determinants of health, which have been shown to influence the incidence of syphilis in historically marginalized racial and ethnic groups [56]. Men who have sex with men (MSM) are also disproportionately affected by syphilis; in 2019, 57% of reported primary and secondary syphilis cases occurred in this population [26]. Of the reported cases of syphilis among MSM, almost half were coinfected with HIV [57]. Our patient fell into all of these high-risk groups to acquire syphilis. Furthermore, the case patient suffered all of the so-called “Big Three” sexually transmitted infections (syphilis, gonorrhea, and chlamydia) [58]. The recent advent of the use of post-exposure doxycycline in persons at high risk for the acquisition of sexually transmitted infections may serve to reduce the burden and transmission of these afflictions [59]. 

In this paper, the appearance of the dramatic rash of malignant syphilis was documented over time. Also, we proposed a new name (ulceronodular-rupioid syphilis (UNRS)) and also found that uncontrolled viremia, and not a low CD4 count, is a major risk factor for HIV-positive patients that manifest with this condition. However, UNRS can occur in patients with other immunocompromising conditions and rarely in persons with no apparent risk factors. Herein, we proposed new diagnostic criteria based on lesion appearance, syphilis serologic status, histopathologic findings, and response to treatment. These new proposed case criteria will provide guidance to clinicians that encounter this severe form of syphilis to properly and efficiently diagnose and treat such patients to prevent the development of additional lesions and complications such as deep organ involvement, superimposed bacterial infections, and subsequent cutaneous scarring.

## Figures and Tables

**Table 1:** Summary of the Characteristics of 75 Patients with Ulceronodular-Rupioid Syphilis.

Characteristic	Wibisono Series (Total N = 45) [7]	This Series (Total N = 30) (Appendix A, Table A1)
Age Range, years; mean age, years	20–86; 44.4	16–61; 35.7
Male, %	84.0	63.3
HIV Infection, %	33/45 (73%)	17/30 (57%)
CD4 Range for HIV Patients	(N = 28); 57–1294 (21 with counts above 200 (75%))	(N = 17); 23–504 (10 with counts above 200 (59%))
Mean CD4 Count, cells/µL	397 (N = 28)	291 (N = 16)
% HIV with Uncontrolled VL	22/27 (81.5%)	15/17 (88.2%)
Time to Presentation (weeks): Range; Mean; Mode	N = 42; 1–36; mean = 7.1; mode = 4	N = 27; 2–20; mean = 6; mode = 4
Other Syphilis Manifestations (number of cases)	neurosyphilis (2), uveitis (1), vitritis (2), keratitis (1), mucus patch (1), condyloma lata (1), osteitis (2), orchitis (1), pulmonary nodules (1)	neurosyphilis (4), uveitis (3), papillitis (1), mucus patch (1), condyloma lata (2), osteitis (1), splenitis (1)
Occurrence of Jarisch–Herxheimer Reaction ^a^	9/38 (24%)	5/17 (29%)
Spirochete Visualization, no. of cases (W-S = Warthin–Starry (silver))	20: no visualization specified; 12/15: immunostain positive (80%); 2/5: W-S or Steiner stains positive (40%)	13: no visualization specified; 10/12: immunostain positive (83%); 2/4: W-S positive (50%); 1 positive by darkfield microscopy
Rx w/Benzathine PCN weekly × 1 dose versus 3 doses; number of cases, %, outcome (improved = improved or resolved)	26 received benzathine PCN only; 5/26 received 1 week (19%); all improved; 21/26 received 3 weeks (81%): 18 improved, 3 lost to follow-up	18 received benzathine PCN only; 5/18 received 1 week (28%); 3 improved, 2 lost to follow-up. 13/18 received 3 weeks (72%): all Improved

^a^ Patients that received prophylactic corticosteroids were omitted; Abbreviation: PCN, penicillin.

**Table 2:** Diagnostic Criteria of Malignant Syphilis from Neisser (1897) and Fisher et al. (1969).

Neisser [15]	Fisher et al. [6]
(1) Relatively short incubation period (2) Constitutional symptoms are pronounced (3) The skin and often mucous membranes of mouth and nose present multiple lesions consisting of large pustules, ulcers, and rupioid ecthymatous lesions (4) May have milder forms of the disease, such as mucous patches, etc. (5) Round or oval pleomorphic skin lesions: papulopustules, ulcerations, ulcers with brown-black rupioid crusts, and healing lesions	(1) Compatible gross and microscopic morphology (2) A high titer serologic test for syphilis (3) Jarisch–Herxheimer reaction (JHR) (4) Dramatic response to antibiotic therapy Gross morphology: similar to Neisser Microscopic morphology: not defined

**Table 3:** Summary of RPR and VDRL titers in the case series of Wibisono [7], the current case series (Table A1), and the Zhu series [30].

	Wibisono et al. [7]	Our Series (Table A1)	Zhu et al. [30]
RPR	(N = 21)	(N = 21)	(N = 26)
Range	4–1024	16–512	32–256
Mean	245	161	140
Median	128	128	128
Mode	256	128	128
Comment	17/21 (81%) had 1:64 or higher	16/21 (76%) had 1:64 or higher	24/26 (92%) had 1:64 or higher
**VDRL**	**(N = 14)**	**(N = 8)**	**(N = 0)**
Range	8–512	16–512	
Mean	179	102	
Median	128	32	
Mode	128	32	
Comment	13/14 (93%) had 1:32 or higher	7/8 (88%) had 1:32 or higher	

**Table 4:** Histopathologic findings in the case series of Wibisono and coworkers [7] and the case series presented herein (Appendix A, Table A1).

Histopathologic Findings	Wibisono et al. (35 Cases) [7]	Our Series (28 Cases)	Both Series (63 Cases)
Plasma cell infiltrate	29/35 (82.9%)	23/28 (82.1%)	52/63 (82.5%)
Lymphohistiocytic infiltrate	23/35 (65.7%)	22/28 (78.6%)	45/63 (71.4%)
Vascular involvement	13/35 (37.1%)	12/28 (42.9%)	25/63(39.7%)
Granulomas	9/35 (25.7%)	8/28 (28.6%)	17/63 (27.0%)
Neutrophilic infiltrate	5/35 (14.2%)	5/28 (17.9%)	10/63 (15.9%)
Giant cells	3/35 (8.6%)	1/28 (3.6%)	4/63 (6.3%)

## Data Availability

The data are contained within the article.

## Appendix A

**Table A1:** Characteristics of Patients with Ulceronodular-Rupioid Syphilis, 2019–2023 ^a,b,c,d^.

Case	Age (years)/ Sex/ [Ref]	HIV Status/ CD4/HIV VL/Underlying Conditions/RPR or VDRL Titer	Description of Lesions/Location/Duration Prior to Presentation/Other Diagnoses	Histopathologic Findings/ Spirochete Visualization by Warthin–Starry (W-S) Stain or Immunostain	Rx; Jarisch–Herxheimer Reaction (JHR) = Yes or No; Outcome
Fi	34/M/ [6]	Pre-HIV era (1967)/ Malnutrition/ RPR 1:256	Ulcerated lesions w/rupioid crusts/leg, R inguinal area, buttock, R dorsal and ventral lower trunk, and R hand/24 weeks/neurosyphilis/*S. aureus* infection	Necrotizing vasculitis; Necrosis of epidermis, upper dermis. Endothelial swelling and proliferation. Perivascular infiltrate in dermis of lymphocytes, plasma cells, some neutrophils; RBC extravasation. Fibrinoid material causing partial to complete lumen obliteration of most of the vessels/Immunostain negative	Tetracycline, 10 days; JHR = No; Resolved over 2 weeks
1	28/M/ This case	HIV-positive/ CD4 411/ VL 35,200/ RPR 1:512	Nodular and ulcerated lesions with rupioid crusts/face, trunk, arms, legs/12 weeks/*S. aureus*, rectal chlamydia, gonorrhea	Lichenoid psoriasiform dermatitis with infiltrate of lymphocytes, plasma cells, and histiocytes; granuloma present/Immunostain positive	IV penicillin × 1 dose; Benzathine PCN weekly × 3 weeks; doxycycline × 17 days; JHR = Yes; Resolved with hyperpigmented scarring
2	26/M/ [5]	HIV-negative/ RPR positive	Multiple ulcerative lesions/legs/8 weeks	Dense infiltrates w/plasma cells and fibrinoid degeneration/Immunostain positive	Minocycline, then amoxicillin × 4 weeks; JHR = No; Outcome not reported
3	29/F/ [60]	HIV-negative/ Hypothyroidism RPR 1:128	Erythematous nodules/scalp, face, neck, axilla, trunk, back, palms and soles, and perineum/6 weeks/fever, myalgias	Subcorneal pustules; dermal granulomas with macrophages, Langerhans cells, lymphocytes, plasma cell infiltrate/Immunostain not specified	IV Benzathine PCN weekly × 3 doses; JHR = No; Resolved over 1 mo
4	43/F/ [61]	HIV-negative/ DM II, Schizophrenia/ RPR 1:128	Nodules and plaques; some ulcerated and crusted/face, neck, trunk, legs, arms/4 weeks	Epidermal acanthosis with diffuse dermal infiltrate of plasma cells/Immunostain positive	IM Benzathine PCN × 1 dose; JHR = No; Outcome not reported
5	33, M/ [62]	HIV-negative/ no PMH/ RPR 1:256	Erythematous-violaceous, ulcerated nodules, and plaques with rupioid crusts/8 weeks	Dermal infiltrate of plasma cells, histiocytes, and lymphocytes with granuloma/Immunostain positive	IV Benzathine PCN weekly × 3; JHR = Yes; Resolved over 6 weeks with hyperpigmented macules
6	44/M/ [63]	HIV-positive/ CD4 86/VL 35,900/RPR 1:32	Blackish-brown lamellated plaques/limbs and scalp/4 weeks	Diffuse dermal lymphocytes and histiocytes admixed with plasma cells/Immunostain not specified	IV ceftriaxone for 2 weeks; JHR = not reported; Improved over 2 weeks
7	41/F/ [64]	HIV-positive/ CD4 164/ VL 223,000/ Alcoholism/ RPR 1:128	Plaques with a peripheral inflammatory-necrotic reaction, raised scabs, and pustular areas with violaceous erythematous background/thorax, extremities, neck, and face/4 weeks/uveitis	Lymphoplasmacytic-histiocytic perivascular and periadnexal dermatitis; epidermis w/pseudo- epitheliomatous hyperplasia, parakeratosis, neutrophilic exocytosis, spongiosis, supra-basal vascular degeneration/Immunostain positive	IV penicillin for 14-days and ciprofloxacin/dexamethasone eye drops for 10-days; JHR = No; Improved
8	48/M/ [43]	HIV-positive/ CD4 88/ VL 495,000/ VDRL 1:32	Erythematous and ulcerated nodules with well-demarcated borders on the face, trunk, and upper arms/8 weeks	Psoriasiform hyperplasia, parakeratosis, dense lichenoid and superficial perivascular lymphoplasmacytic infiltrate/Immunostain positive	Doxycycline for 4 weeks; JHR = No; Resolved
9	21/F/ [65]	HIV-positive/ CD4 23/ VL 89,125/ RPR 1:16	Erythematous papules and plaques, some with a central necrotic eschar/trunk, arms/3 weeks/none	Not performed	Benzathine PCN IM × 1 dose; JHR = Yes; Resolved within a month with residual scarring
10	47/M/ [66]	HIV-positive/ CD4 320/ VL 60,500/ RPR 1:256	Eroded plaques/cheek and forearm/2 weeks/neurosyphilis, R eye uveitis, L eye papillitis	Psoriasiform and vacuolar interface reaction w/superficial and deep dermal perivascular/periadnexal lymphoplasmacytic infiltrate dominated by plasma cells/Immunostain positive	IV benzyl PCN, 15-d and corticosteroids; JHR = No (corticosteroids); Full recovery after 2 mos
11	26/F/ [17]	HIV-negative/ Modified VDRL 1:128	Erythematous scaly plaques/forehead, jaw eyelids, axilla, fingers, anogenital area/2 weeks	Plasma cell-rich granulation tissue/Immunostain not described	IM Benzathine PCN weekly × 3; JHR = Yes; resolved after 2 weeks
12	55/M/ [67]	HIV-negative/ alcohol abuse/ RPR 1:128	Ulcers with rupioid crusts/face, scalp, trunk, extremities/2 weeks/myiasis	Epidermal hyperplasia and infiltration with plasma cells and lymphocytes in dermis/Immunostain not described	IM benzathine PCN weekly × 3; JHR = No (corticosteroids); Resolved in one mo, with scarring
13	35/M/ [68]	HIV-positive CD4 291/ VL untreated/ RPR 1:128	Nodules and ulcers with thick brown-black crusts/axilla, trunk, back, inguinal, penis, soles/4 weeks	Epidermal hyperkeratosis and acanthosis. In the dermis, lymphocytes, and histiocytes and dense perivascular and periadnexal plasma cells/Imunostain not described	IM benzathine PCN weekly × 3; JHR = No; Resolved over 3 weeks with hyperpigmented scarring
14	41/F/ [69]	HIV-negative/ malnutrition, alcoholic hepatitis/ RPR 1:128	Plaques with crusts and erosions on nose, cheeks, neck, scalp, trunk, limbs/12 weeks/tonsillar mucus patch/*S. aureus*	Dermal infiltrate of atypical T-cells, numerous plasma cells, and histiocytes/W-S stain positive	IV benzylpenicillin for 14 days; JHR = No (cortico-steroids); Lesions resolved, mild hyperpigmented scarring
15	30/M/ [70]	HIV-negative/ healthy/ RPR 1:32	Oyster shell-like skin lesions on his scalp, face, trunk, arms, and legs/4 weeks/condyloma lata	Dense infiltrate of lymphocytes, plasma cells, and neutrophils in the dermis/W-S stain positive	IM benzathine PCN weekly × 3 doses; JHR = No; Resolution within 3 weeks without scarring
16	28/F/ [71]	HIV-negative/ baseline health not stated/ RPR 1:128	Ulcerated papules and plaques involving the face, shins, knees, and thighs/6 weeks/uveitis	Lichenoid granulomatous dermatitis with plasma cells/ Immunostain not described	IV penicillin G for 14 days; JHR not specified; Outcome not described
17	61/M/ [72]	HIV-positive/(‘poorly controlled’; no CD4, VL stated)/ RPR 1:128	Oval plaques with central necrosis, crust, and ulceration w/surrounding erythema/distribution not described/3 weeks/arthalgia	Abscess w/blood vessels with fibrinoid necrosis of vessel wall and neutrophilic infiltrate in and around the vessel/Immunostain negative	IV penicillin for 4 weeks; JHR not specified; Resolution over one month
18	22/M/ [19]	HIV-positive/ CD4 117/ VL 420,000/ RPR 1:64	Maculopapules/blisters on face, trunk, limbs; treated w/levofloxacin w/o improvement; blisters ruptured; skin ulcerated and scabbed with brown–black rupioid crusts/4 weeks	Dense infiltration of dermis w/neutrophils, lymphocytes, and histocytes; perivascular infiltration of lymphocytes and plasma cells/Immunostain not described	IM Benzathine PCN weekly × 3 doses; JHR not specified; rash resolved. Re-infected with syphilis 13 mos later; similar rash, RPR 1:128 (CD4 657); doxycycline for 4 weeks, rash resolved
19	24/M/ [42]	HIV-positive/ CD4 470/ VL untreated VDRL 1:512	Ulcers with rupioid surface/face, trunk, inguinal area, arms/8 weeks/Condyloma lata	Spongiotic dermatitis w/many dermal neutrophils; endarteritis, and microthrombi in blood vessels/Darkfield positive	IM Benzathine PCN IM X 1 dose; JHR = No; resolved in one month with hyperpigmentation
20	57/M/ [73]	HIV-positive/ CD4 504/ VL < 20 RPR 1:128	Ulcerated plaques and nodules, with lamellar crusts/scalp, face, trunk, limbs; 4 weeks	Lymphohistiocytic infiltrate in superficial dermis, no plasma cells/Immunostain positive	IM Benzathine PCN weekly × 3 weeks; JHR = No; Resolved with hypopigmented scarring
21	35/M/ [74]	HIV-negative/ Hepatitis B/ RPR 1:16	Ulcerative lesions with hemorrhagic crusts/trunk, extremities, genitals/ 3 weeks	Parakeratosis, acanthosis, prominent spongiosis, lymphocyte exocytosis, and dermal lymphohistiocytic infiltrate and perivascular, periadnexal, perineural plasma cells/Immunostain not stated	IM Benzathine PCN weekly × 3 weeks; JHR = No; Improvement within 4-days after first dose
22	42/M/ [75]	HIV-positive/ CD4 399/ VL 102,000/ RPR 1:512	Ulcers w/keratosis and crusting/scalp, face, perineum, limbs/4 weeks/neurosyphilis	Dermal infiltrate of plasma cells, lymphocytes, and histiocytes, in a lichenoid pattern with psoriasiform hyperplasia/Immunostain positive	IV PCN; JHR not specified; Lost to f/u
23	31/M/ [76]	HIV-positive/ CD4 481/ VL 89,200 Hepatitis C, IV drug abuse/ RPR 1:256	Ulcerated papules plaques w/purulent and sanguineous drainage and adherent crusts/trunk, limbs, scalp, face/“weeks old”/*S. aureus*	Suppurative granulomatous and lymphoplasmacytic inflammation w/overlying lichenoid and spongiotic dermatitis/Immunostain negative	IM Benzathine PCN × 1 dose; JHR not specified; Lost to follow-up
24	22/M/ [77]	HIV-positive/ CD4 284/ VL 243,000/ VDRL 1:32	Erythematous and ulcerated plaques (some with necrotic crusts)/trunk, arms, legs/4 weeks	Not performed	IM Benzathine PCN × 1 dose; JHR not specified;Complete resolution
25	50/F/ [78]	HIV-positive CD4 338/ VL undetected/ VDRL 1:32	Ulcers, plaques and nodules/neck, face, arm, thigh/12 weeks	Granulomas w/neutrophils, histiocytes, lymphocytes, plasma cells; dermal vessels w/endothelial swelling. fibrinoid necrosis and leukocytoclasia in a small dermal vessel/W-S negative/Immunostain positive	IM Benzathine PCN weekly × 3 doses; JHR = No; Resolved with hyperpigmented macules
26	16/F/ [79]	HIV negative/ JRA, on Immunosupp./ VDRL 1:32	Necrotic ulcers/face, trunk, arms, legs, hands, palms, oral mucosa/duration not stated	Superficial and deep vacuolar interface dermatitis with many plasmocytes/W-S stain negative	IM Benzathine PCN weekly × 3 weeks; JHR = No; Complete resolution
27	56/M/ [80]	HIV-negative/ Malnutrition/ RPR 1:16	Ulcerated and necrotic papules and nodules with black crusts/face, trunk, extremities, genitals/8 weeks/neurosyphilis	Obliterative vasculitis; dermal infiltrate of lymphocytes and plasma cells/Immunostain positive	IV PCN × 11-d; JHR = No (corticosteroids); then benzathine PCN weekly × 3-weeks; Resolved w/hyperpigmented scarring
28	29/F/ [20]	HIV-negative Crohn’s disease, on adalimumab/ VDRL 1:16	Ulcerated papules and nodules with crusts that became rupioid/face, neck, trunk, arms/duration not stated	Epidermal ulcers, basal cell degeneration, dermal lymphohistiocytosis; vessels w/reactive endothelial changes/Immunostain not done	IM Benzathine PCN weekly × 3 doses; JHR = Yes; Resolved with hypopigmented scarring
29	22/M/ [81]	HIV-positive/ CD4 236/ VL uncontrolled/ RPR 1:256	Palpable purpura on legs followed by nodules and ulcers w/thick crusts/face, trunk, and arms/3 weeks/leukocytoclastic vasculitis	Superficial and perivascular infiltrate of plasma cells, lymphocytes; central ulcer w/psoriasiform hyperplasia. Dermal, perivascular plasma cells, lymphocytes, and histiocytes/Immunostain not done	IM Benzathine PCN weekly × 3 doses; JHR = No (corticosteroids); Resolved with hyperpigmented macules
30	30/F/ [4]	HIV-positive/ CD4 444/ VL untreated/ Cachexia/ VDRL 1:32	Ulcers with ecthymatous crusts/back, abdomen, limbs, genitals/20 weeks/neurosyphilis, splenic abscesses, osteitis/vaginal candidiasis	Acanthosis, spongiosis; dermal edema with peri-vascular and peri-appendageal infiltrate of plasma cells, lymphocytes/Immunostain not done	IV PCN × 15-days; JHR not specified; resolved over 2 weeks with hypopigmented scarring

^a^ CD4 in cells/µL. ^b^ HIV viral load in virions/mL. ^c^ A glossary of dermatopathology terms is located in Appendix B. ^d^ Abbreviations: JRA, juvenile rheumatoid arthritis; PCN, penicillin; VL, HIV viral load; neg, negative; Immunosupp., immunosuppressives (tocilizumab, prednisone, methotrexate); *S. aureus*, *Staphylococcus aureus* skin and soft tissue infection.

## Appendix B. Glossary of Dermatopathology Terms

**Acanthosis**—an increase in the thickness of the stratum spinosum of the epidermis [82]
**Adnexal**—referring to skin appendages (hair follicles; sebaceous/apocrine/eccrine glands) [82]
**Spongiosis**—inflammatory intercellular edema of the epidermis [82]
**Leukocytoclasia**—karyorrhexis of leukocytes [82]
**Lichenoid**—a band-like infiltration of inflammatory cells in the superficial dermis, parallel to the epidermis [82]
**Parakeratosis**—mode of keratinization with retention of nuclei in the cells of the stratum corneum of the epidermis, observed in many scaling dermatoses, such as psoriasis [82]
**Psoriasiform**—epidermal hyperplasia with elongation of the rete ridges and dermal papillae in a regular manner [83]
